# Significance of Detecting Serum Antibodies to Outer Surface Protein A of Lyme Disease *Borreliae* in PCR-Confirmed Blood Infections

**DOI:** 10.3390/diagnostics14232704

**Published:** 2024-11-30

**Authors:** Jyotsna S. Shah, Ranjan Ramasamy

**Affiliations:** 1 IDFISH Technology Inc., Milpitas, CA 95035, USA; 2 IGeneX, Milpitas, CA 95035, USA

**Keywords:** antibodies to OspA, *Borrelia burgdorferi*, borreliosis, immunoblots, immunodiagnosis, Lyme disease, outer surface protein A, protective immunity, Lyme disease *Borreliae*, Lyme disease vaccines

## Abstract

**Background/Objectives**: Lyme disease is caused by some species of tick-borne bacteria of the genus *Borrelia*, termed Lyme disease *Borreliae* (LDB). *Borrelia burgdorferi* is the LDB species principally responsible for Lyme disease in the US. The outer surface protein A (OspA) of LDB attaches the bacteria to the gut of *Ixodes* tick vectors. OspA expression is downregulated when *B. burgdorferi* is transmitted from ticks to mammalian hosts. Vaccination with OspA elicits antibody-mediated protective immunity in animals and humans against LDB infection. The possible presence of serum antibodies against OspA in persons with PCR-confirmed LDB infections in blood was investigated in this study. **Methods**: Ninety-one archived sera from patients with LDB infections in blood demonstrated by a sensitive PCR assay were tested for reactivity with OspA from multiple LDB species in line immunoblots. **Results**: In total, 14 of the 91 sera (15.4%) had either IgG or IgM antibodies to OspA from one or more LDB species. **Conclusions**: The results show for the first time that serum antibodies to OspA are formed when LDB are present in human blood. However, the factors that governed the expression of OspA by LDB in patients could not be ascertained. It will be useful to determine whether the observed levels of serum antibodies to OspA in infected persons can protect against subsequent tick-borne infection and whether OspA used in conjunction with other LDB antigens can improve the serological diagnosis of Lyme disease.

## 1. Introduction

Lyme disease is a tick vector-borne disease caused by several species of spirochete bacteria of the genus *Borrelia*, termed Lyme disease *Borreliae* (LDB). The disease is mainly prevalent in the temperate climate zone [1,2]. The best-studied LDB species responsible for Lyme disease in humans in the US is *Borrelia burgdorferi* [1,2]. Lyme disease in Europe is caused by *B. garinii*, *B. afzellii* and *B. burgdorferi* [1,2]. However, other LDB species such as *B. spielmanii*, *B. californiensis*, *B. bissettii*, *B. mayonii* and *B. carolinensis* in the US and *B. valaisiana*, *B. lusitaniae* and *B. spielmanii* in Europe have also been reported to be capable of infecting humans [2,3,4]. The principal tick vectors of Lyme disease are *Ixodes scapularis* and *I. pacificus* in the US, and *I. ricinus* and *I. persulcatus* in Europe. The ticks acquire LDB from infected wild mammals such as deer and mice. After a period of development in the tick gut, the spirochetes are transmitted to humans via the salivary glands during blood feeding [2]. The detection of LDB by microscopy and culturing from patient tissues usually has poor diagnostic sensitivity and specificity and is also time-consuming and labor-intensive. PCR-based direct detection of LDB in blood is often insensitive, due to a low concentration of spirochetes in the blood [5], the presence of PCR inhibitors in the blood and sequestration of the spirochetes in tissues other than blood [5,6,7]. Therefore, the clinical diagnosis of Lyme disease mainly relies on the laboratory confirmation of infection by detecting serum antibodies to LDB antigens [1,2,5,8]. In the US, a standard two-tier test procedure for detecting serum antibodies was first established by the US Centers for Disease Control and Prevention (CDC) in 1995 [8]. The first-tier test was performed with sera on whole *B. burgdorferi* cell lysates by an enzyme immunoassay or *B. burgdorferi* cells by an immunofluorescence assay [8]. A positive or equivocal result in a first-tier test required a positive second-tier Western blot test for detecting IgM and IgG antibodies against a panel of *B. burgdorferi* antigens [8]. This widely used standard two-tier test approach for detecting serum antibodies in the US [8] has been recently supplemented with a variety of modified two-tier tests. The modified two-tier tests utilize sequential enzyme immunoassays against different *B. burgdorferi* antigens or a combination of enzyme immunoassay and recombinant *B. burgdorferi* proteins as target antigens in immunoblots [1,2,5].

The outer membrane of LDB contains several outer surface proteins termed Osps. One of the Osps, the 31 kDa OspA, the gene for which is located on a linear plasmid found in LDB, contains a covalently linked lipid molecule that anchors OspA to the spirochete membrane [9]. OspA is not included in the panel of antigens used for the second-tier Western blot tests recommended by the CDC, which includes only one Osp, viz. the 23 kDa OspC, as a target antigen [8]. The structure of OspA has been well characterized to the extent that its crystallographic structure in complex with antibodies has been determined [10,11]. OspA has the essential function of binding LDB to a specific receptor protein in the gut of the tick vector from where the spirochetes are subsequently transferred through the tick salivary glands to mammalian hosts when the tick takes a blood meal [12,13,14]. Experiments with OspA knock-outs have shown that OspA is not essential for LDB to produce an infection in mammalian hosts [15]. This is consistent with the downregulation of the expression of OspA during the transmission of LDB from ticks to mammalian hosts [14,15].

Vaccination with OspA has been shown to protect experimental animals against tick-borne LDB infection through a mechanism that involves anti-OspA antibodies entering ticks during blood feeding and then neutralizing OspA-expressing LDB within ticks [16]. Recombinant OspA proteins are also being developed for vaccinating humans against tick-borne infections with LDB, but they are not yet available for human use [17,18,19]. However, commercially available OspA-based vaccines are successfully used to protect dogs against Lyme disease caused by LDB acquired from infected ticks [17,20].

The present study was designed to determine whether antibodies to OspA are formed when LDB are present in patients’ blood because OspA expression is downregulated during mammalian infection [14,15], and antibodies to OspA protect against infection with LDB [16,18]. For this purpose, we tested patient sera with line immunoblots incorporating OspA antigens from multiple LDB species as well as other LDB target antigens [21]. The presence of LDB in blood was confirmed by a sensitive and specific PCR procedure developed at IGeneX termed the Lyme multiplex PCR-dot blot assay [22]. This assay is sensitive because it first concentrates and purifies LDB DNA from blood using a set of LDB-specific capture DNA probes and magnetic separation before PCR amplification is performed and the amplified DNA is detected in dot blots with specific DNA probes [22]. The sensitivity of the Lyme multiplex PCR-dot blot assay is such that only 17.4% of PCR-positive patient blood samples were positive on the standard two-tier serological test recommended by the CDC [22].

## 2. Materials and Methods

### 2.1. Patient Samples

IGeneX (https://igenex.com, accessed on 27 November 2024) is a fully certified clinical diagnostic laboratory that provides serological and molecular diagnostic tests for Lyme disease and other tick-borne diseases. Serum and whole blood samples from patients with suspected Lyme disease are routinely received at IGeneX for the laboratory confirmation of Lyme disease following a referral by physicians. However, the referring physicians do not provide IGeneX with clinical details of the patients. Surplus sera that are left over after testing are sometimes archived and stored at −80 °C at IGeneX. Archived IGeneX serum samples from 91 patients who had tested positive for blood-stage LDB in the IGeneX Lyme multiplex PCR-dot blot assay in a previous study [22] were used in the present investigation. The 91 serum samples were analyzed for IgG and IgM antibodies to OspA with the IGeneX Lyme disease IgG and IgM line immunoblots described in Section 2.2.

### 2.2. Lyme Disease Immunoblots

Lyme disease immunoblot tests for detecting serum IgG and IgM antibodies to the 31 kDa OspA were performed as previously described [21] with minor modifications. Recombinant OspA proteins derived from nine different US and European LDB species were applied as separate lines on nitrocellulose membranes in the immunoblots. The same Lyme immunoblots are also used for routine testing of other patient sera for Lyme disease at IGeneX. Therefore, they also incorporated other LDB target antigens besides OspA and specific control proteins [21]. IgG immunoblots incorporated the following antigen bands in kDa in addition to the 31 kDa OspA band: 18, 23 (OspC), 28, 30, 34 (OspB), 39 (BmpA), 41 (FlaB), 45, 58, 66 and 93 [21]. IgM immunoblots had the following antigen bands in kDa in addition to the 31 kDa OspA band: 93, 41 (FlaB), 39 (BmpA), 34 (OspB) and 23 (OspC). The 23 (OspC), 39 (BmpA) and 41 (FlaB) antigens, like 31 (OspA), also contained proteins derived from multiple LDB species. Additionally, both IgG and IgM immunoblots included a target antigen termed LSA, which was a chimeric C6 peptide from the immunodominant region of the variable surface protein VlsE derived from multiple LDB species [21]. Two control proteins—C1, a mix of human IgG and IgM for confirming the addition of alkaline phosphatase conjugated anti-human immunoglobulin antibodies, and C2, Protein L for confirming the addition of human sera—were also applied as lines in the immunoblots [21]. Protein L also served as an internal calibration control, so that any band found having a visual intensity equal to or greater than the C2 control band intensity was considered to be a positive band. A mixture of human sera from patients with confirmed Lyme disease was used as a positive control (P) and sera from uninfected persons as a negative control (N).

### 2.3. Ethical Considerations

US Government Regulation CFR 46.104 (d) (4) (ii) permits the retrospective analysis of de-identified clinical test results, and the use of leftover de-identified sera without patient consent and institutional review board approval. All methods were performed in accordance with the Declaration of Helsinki and CFR 46.104 (d) (4) (ii).

## 3. Results

**Figure 1:**
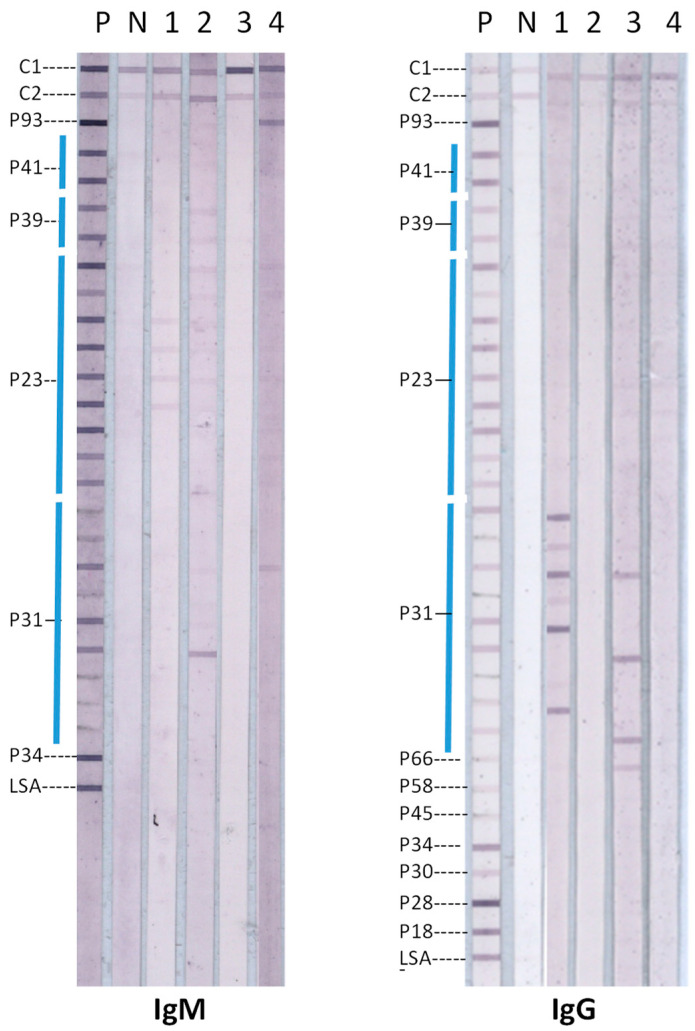
**Examples of IgM and IgG line Lyme disease immunoblots with sera containing antibodies to OspA**. Target antigens are labelled based on the relative molecular mass of their parent proteins, e.g., P31 is OspA, as previously described [21]. OspA (P31) proteins from nine different LDB species used as target antigens were applied as separate lines on the nitrocellulose membrane. Information on other target antigens, control proteins C1 and C2, as well as the positive (P) and negative (N) control blots are given in the Methods Section 2.2.

A proportion of the 91 sera from patients with PCR-confirmed LDB in blood had IgG or IgM antibodies against the 31 kDa OspA that were detected by the IGeneX Lyme immunoblots. Representative examples of IgM and IgG immunoblots of four patient sera with either IgG or IgM antibodies that bound to OspA target antigens in the immunoblots are shown in Figure 1.

The immunoblots in Figure 1 show that sera 2 and 4 possessed IgM antibodies, and sera 1 and 3 possessed IgG antibodies, reacting with one or more OspA antigen bands in the immunoblots. The four sera that recognized OspA in the immunoblots reacted variably with OspA antigens from different LDB species (Figure 1). This suggests that the anti-OspA antibodies in the sera may have been formed as a consequence of infection with different LDB species. However, this needed to be confirmed by DNA sequencing of LDB in patient samples, which was not possible in the present study. Patient sera also reacted with LDB antigens other than OspA in the immunoblots (Figure 1). The OspA reactivity of all 91 sera from patients with PCR-confirmed *B. burgdorferi* blood infection are summarized in Table 1.

Because IgG and IgM antibodies against OspA did not occur together in any one of the 91 sera, the proportion of tested sera with either IgG or IgM antibodies to OspA was 14/91 (15.4%). The difference between the proportions of sera containing IgG and IgM antibodies to OspA was not statistically significant according to the Chi-Square test with Yates correction (χ^2^ = 0.7, *p* = 0.4).

## 4. Discussion

For the first time, a small proportion of patients with active blood-stage spirochetemia are shown to produce IgG and IgM antibodies to OspA that can be detected by immunoblots. Further investigations are needed to elucidate how antibodies against OspA are formed when LDB are present in blood because OspA expression is reported to be normally downregulated in mammalian hosts [15,16,23,24]. It is possible that residual OspA exposed on the surface of spirochetes inoculated by ticks during a bloodmeal or synthesized at low concentrations during their subsequent growth and replication in blood and other tissues elicit the formation of IgM and IgG antibodies to OspA in some patients. It is relevant in this context that *B. burgdorferi* cultured in vitro at 37 °C is able to synthesize OspA [25]. Furthermore, rhesus macaque monkeys, which are a good model for human Lyme disease, have been shown to generate antibodies against OspA when they are inoculated by needle and syringe with in vitro cultured *B. burgdorferi* [26].

Several vaccines for Lyme disease that are based on whole LDB cell lysates or recombinant OspA protein are presently available for veterinary use [17,20]. OspA produced through recombinant DNA technology and subsequently chemically linked to a lipid before being formulated in an alum adjuvant became the first and, so far, the only human vaccine (termed LYMErix^®^) approved for Lyme disease by the US Food and Drug Administration [17]. Although LYMErix^®^ had shown significant antibody-dependent protection in Phase 3 human trials in the US [18], its marketing was discontinued by the manufacturer in 2002. Several serotypes of OspA have been identified in different LDB species [17]. A recent experimental Lyme disease vaccine, termed VLA15, based on the more common US and European LDB OspA serotypes, has been shown to protect mice against infection with different LDB species [19]. VLA15 is composed of three fusion proteins, each corresponding to the C-termini of two OspA serotypes with a lipid moiety covalently linked to the N-termini, and which is then formulated with aluminum hydroxide as an adjuvant [19]. Other types of OspA-based vaccines for human use are also being developed [27,28,29]. VLA-15 recently demonstrated a satisfactory safety profile and immunogenicity after primary and boosting immunizations in a controlled multi-center phase II clinical trial [30] and is expected to progress to clinical trials for testing protective efficacy against LDB infection.

Since antibodies to OspA protect against Lyme disease [18], immunoblots and other serological tests with OspA may be useful for assessing the levels of protection in persons vaccinated with OspA-based protein vaccines. Because little is known about acquired immunity in Lyme disease, OspA antibody tests may also help in assessing protective immunity against a subsequent LDB infection in unvaccinated persons who have recovered from Lyme disease.

A pilot study with immunoblots that utilized OspA as one of the scoring target antigens for detecting IgG and IgM antibodies to LDB in OspA-unvaccinated persons with Lyme disease symptoms yielded sensitivities and specificities of detection that were comparable to the CDC standard two-tier test for Lyme disease [21]. This also applied to a small number of patients in this study with acute or early localized infection (stage 1 Lyme disease), early disseminated Lyme disease with neuroborreliosis (stage 2), or late disseminated Lyme disease with arthritis (stage 3) [21]. Therefore, a larger controlled study to investigate the potential use of OspA as one of a panel of scoring target antigens in the serodiagnosis of Lyme disease is also warranted.

## Figures and Tables

**Table 1:** Sera with IgG and IgM antibodies to OspA in PCR-confirmed infections.

	IgG Antibodies Alone	IgM Antibodies Alone	Both IgG and IgM Antibodies
No. of positive sera	9	5	0
Percent positive of the 91 sera tested	9.9%	5.5%	0%

## Data Availability

All data needed for interpretation of the results and arriving at the conclusions are provided in the manuscript.

## References

[1] Centers for Disease Control and Prevention Lyme Disease. https://www.cdc.gov/lyme/index.html.

[2] Shah J.S., Burrascano J.J., Ramasamy R. (2023). Recombinant protein immunoblots for differential diagnosis of tick-borne relapsing fever and Lyme disease. J. Vector Borne Dis..

[3] Mathiesen D.A., Oliver J.H., Kolbert C.P., Tullson E.D., Johnson B.J., Campbell G.L., Mitchell P.D., Reed K.D., Telford S.R., Anderson J.F. (1997). Genetic heterogeneity of *Borrelia burgdorferi* in the United States. J. Infect. Dis..

[4] Wilhelmsson P., Fryland L., Börjesson S., Nordgren J., Bergström S., Ernerudh J., Forsberg P., Lindgren P.E. (2010). Prevalence and diversity of *Borrelia* species in ticks that have bitten humans in Sweden. J. Clin. Microbiol..

[5] Branda J.A., Steere A.C. (2021). Laboratory diagnosis of Lyme borreliosis. Clin. Microbiol. Rev..

[6] Hyde J.A. (2017). *Borrelia burgdorferi* Keeps moving and carries on: A review of borrelial dissemination and invasion. Front. Immunol..

[7] Tracy K.E., Baumgarth N. (2017). *Borrelia burgdorferi* manipulates innate and adaptive immunity to establish persistence in rodent reservoir hosts. Front. Immunol..

[8] Centers for Disease Control and Prevention (1995). Notice to readers: Recommendations for test performance and interpretation from the second national conference on serologic diagnosis of Lyme disease. Morb. Mortal. Wkly. Rep..

[9] Barbour A.G. (1988). Plasmid analysis of *Borrelia burgdorferi*, the Lyme disease agent. J. Clin. Microbiol..

[10] Li H., Dunn J.J., Luft B.J., Lawson C.L. (1997). Crystal structure of Lyme disease antigen outer surface protein A complexed with a Fab. Proc. Natl. Acad. Sci. USA.

[11] Rudolph M.J., Davis S.A., Haque H.M.E., Ejemel M., Cavacini L.A., Vance D.J., Willsey G.G., Piazza C.L., Weis D.D., Wang Y. (2023). Structure of a transmission blocking antibody in complex with outer surface protein A from the Lyme disease spirochete, *Borreliella burgdorferi*. Proteins.

[12] Pal U., de Silva A.M., Montgomery R.R., Fish D., Anguita J., Anderson J.F., Lobet Y., Fikrig E. (2000). Attachment of *Borrelia burgdorferi* within *Ixodes scapularis* mediated by outer surface protein A. J. Clin. Investig..

[13] Yang X.F., Pal U., Alani S.M., Fikrig E., Norgard M.V. (2004). Essential role for OspA/B in the life cycle of the Lyme disease spirochete. J. Exp. Med..

[14] Pal U., Li X., Wang T., Montgomery R.R., Ramamoorthi N., De Silva A.M., Bao F., Yang X., Pypaert M., Pradhan D. (2004). TROSPA, an *Ixodes scapularis* receptor for *Borrelia burgdorferi*. Cell.

[15] Schwan T.G., Piesman J. (2000). Temporal changes in outer surface proteins A and C of the Lyme disease-associated spirochete, *Borrelia burgdorferi*, during the chain of infection in ticks and mice. J. Clin. Microbiol..

[16] de Silva A.M., Telford S.R., Brunet L.R., Barthold S.W., Fikrig E. (1996). *Borrelia burgdorferi* OspA is an arthropod-specific transmission-blocking Lyme disease vaccine. J. Exp. Med..

[17] O’Bier N.S., Hatke A.L., Camire A.C., Marconi R.T. (2021). Human and veterinary vaccines for Lyme disease. Curr. Issues Mol. Biol..

[18] Steere A.C., Sikand V.K., Meurice F., Parenti D.L., Fikrig E., Schoen R.T., Nowakowski J., Schmid C.H., Laukamp S., Buscarino C. (1998). Vaccination against Lyme disease with recombinant *Borrelia burgdorferi* outer-surface lipoprotein A with adjuvant. Lyme Disease Vaccine Study Group. N. Engl. J. Med..

[19] Comstedt P., Schueler W., Meinke A., Lundberg U. (2017). The novel Lyme borreliosis vaccine VLA15 shows broad protection against *Borrelia* species expressing six different OspA serotypes. PLoS ONE.

[20] Camire A.C., Hatke A.L., King V.L., Millership J., Ritter D.M., Sobell N., Weber A., Marconi R.T. (2021). Comparative analysis of antibody responses to outer surface protein (Osp)A and OspC in dogs vaccinated with Lyme disease vaccines. Vet. J..

[21] Liu S., Cruz I.D., Ramos C.C., Taleon P., Ramasamy R., Shah J. (2018). Pilot Study of immunoblots with recombinant *Borrelia burgdorferi* antigens for laboratory diagnosis of Lyme disease. Healthcare.

[22] Shah J.S., D’Cruz I., Ward S., Harris N.S., Ramasamy R. (2018). Development of a sensitive PCR-dot blot assay to supplement serological tests for diagnosing Lyme disease. Eur. J. Clin. Microbiol. Infect. Dis..

[23] Woodman M.E., Cooley A.E., Stevenson B. (2008). Production of outer surface protein A by *Borrelia burgdorferi* during transmission from infected mammals to feeding ticks is insufficient to trigger OspA seroconversion. FEMS Immunol. Med. Microbiol..

[24] Wagner B., Freer H., Rollins A., Garcia-Tapia D., Erb H.N., Earnhart C., Marconi R., Meeus P. (2012). Antibodies to *Borrelia burgdorferi* OspA, OspC, OspF, and C6 antigens as markers for early and late infection in dogs. Clin. Vaccine Immunol..

[25] Ramamoorthy R., Philipp M.T. (1998). Differential expression of *Borrelia burgdorferi* proteins during growth in vitro. Infect. Immun..

[26] Embers M.E., Hasenkampf N.R., Jacobs M.B., Philipp M.T. (2012). Dynamic longitudinal antibody responses during *Borrelia burgdorferi* infection and antibiotic treatment of rhesus macaques. Clin. Vaccine Immunol..

[27] O’Bier N.S., Camire A.C., Patel D.T., Billingsley J.S., Hodges K.R., Marconi R.T. (2024). Development of novel multi-protein chimeric immunogens that protect against infection with the Lyme disease agent *Borreliella burgdorferi*. mBio.

[28] Kamp H.D., Swanson K.A., Wei R.R., Dhal P.K., Dharanipragada R., Kern A., Sharma B., Sima R., Hajdusek O., Hu L.T. (2020). Design of a broadly reactive Lyme disease vaccine. NPJ Vaccines.

[29] Pavia C.S., Saggio G., Plummer M.M. (2024). The major epidemiologic, microbiologic, immunologic, and clinical aspects of Lyme disease that form the basis for a newly developed vaccine that may become available soon for human use. Front. Immunol..

[30] Ghadge S.K., Schneider M., Dubischar K., Wagner L., Kadlecek V., Obersriebnig M., Hochreiter R., Klingler A., Larcher-Senn J., Derhaschnig U. (2024). Immunogenicity and safety of an 18-month booster dose of the VLA15 Lyme borreliosis vaccine candidate after primary immunization in healthy adults in the USA: Results of the booster phase of a randomised, controlled, phase 2 trial. Lancet Infect. Dis..

